# Metabolic Regulation of Neuronal Plasticity by the Energy Sensor AMPK

**DOI:** 10.1371/journal.pone.0008996

**Published:** 2010-02-01

**Authors:** Wyatt B. Potter, Kenneth J. O'Riordan, David Barnett, Susan M. K. Osting, Matthew Wagoner, Corinna Burger, Avtar Roopra

**Affiliations:** Department of Neurology, University of Wisconsin-Madison, Madison, Wisconsin, United States of America; INSERM U862, France

## Abstract

Long Term Potentiation (LTP) is a leading candidate mechanism for learning and memory and is also thought to play a role in the progression of seizures to intractable epilepsy. Maintenance of LTP requires RNA transcription, protein translation and signaling through the mammalian Target of Rapamycin (mTOR) pathway. In peripheral tissue, the energy sensor AMP-activated Protein Kinase (AMPK) negatively regulates the mTOR cascade upon glycolytic inhibition and cellular energy stress. We recently demonstrated that the glycolytic inhibitor 2-deoxy-D-glucose (2DG) alters plasticity to retard epileptogenesis in the kindling model of epilepsy. Reduced kindling progression was associated with increased recruitment of the nuclear metabolic sensor CtBP to NRSF at the BDNF promoter. Given that energy metabolism controls mTOR through AMPK in peripheral tissue and the role of mTOR in LTP in neurons, we asked whether energy metabolism and AMPK control LTP. Using a combination of biochemical approaches and field-recordings in mouse hippocampal slices, we show that the master regulator of energy homeostasis, AMPK couples energy metabolism to LTP expression. Administration of the glycolytic inhibitor 2-deoxy-D-glucose (2DG) or the mitochondrial toxin and anti-Type II Diabetes drug, metformin, or AMP mimetic AICAR results in activation of AMPK, repression of the mTOR pathway and prevents maintenance of Late-Phase LTP (L-LTP). Inhibition of AMPK by either compound-C or the ATP mimetic ara-A rescues the suppression of L-LTP by energy stress. We also show that enhanced LTP via AMPK inhibition requires mTOR signaling. These results directly link energy metabolism to plasticity in the mammalian brain and demonstrate that AMPK is a modulator of LTP. Our work opens up the possibility of using modulators of energy metabolism to control neuronal plasticity in diseases and conditions of aberrant plasticity such as epilepsy.

## Introduction

Long Term Potentiation (LTP) is thought to represent one form of durable alteration in synaptic strength underlying memory formation[1]. Despite this potential role in learning and memory, the ability to control aberrant LTP formation under pathological conditions may be of therapeutic value. The initiating events in LTP expression are rapid and require neither de-novo protein synthesis nor transcription (termed Early LTP–E-LTP), however LTP maintenance requires both protein translation and mRNA transcription (Late LTP–L-LTP) [2], [3], [4], [5]. This protein synthesis appears to be dependent on mammalian Target of Rapamycin (mTOR), a kinase complex that phosphorylates and activates key positive regulators of protein translation including p70S6K kinase, which then further phosphorylates the downstream target, ribosomal protein S6 (rpS6) [6], [7]. These downstream effectors act to increase translation of select mRNAs that enhance overall translational capacity [6], [7], [8].

**Figure 1:**
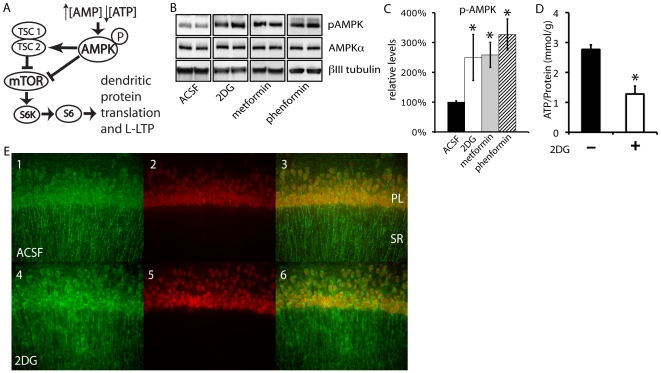
Metformin and 2DG activate AMPK in hippocampal CA1 neurons. A) Schematic of the AMPK-mTOR pathway. B) AMPK is activated 30 min after exposure to 2DG (10 mM, p = 0.019, n = 9), metformin (5 µM, p = 0.005, n = 6), or phenformin (10 µM, p = 0.018, n = 6). Hippocampal slices were incubated in ACSF and drug for 30 minutes and subjected to western blot with anti-phospho-Thr172-AMPK antibody followed by βIII-tubulin as a loading control. Representative western blots of duplicate lanes are shown, together with their quantification from at least 10 samples per condition (C). D) ATP levels are reduced in the presence of 10 mM 2DG. Slices were incubated in ACSF+10 mM 2DG (n = 3) as above. Tissue was lysed and subjected to a CellTiter-Glo ATP assay (Promega). E) 2DG activates AMPK in cell bodies of the pyramidal layer (PL) and dendrites of the stratum radiatum (SR). Anti-phospho-Thr172-AMPK immunoreactivity is displayed in green. Neu-N is displayed in red. Scale bar: 10 µm.

In non-neuronal systems the mTORC1 complex of mTOR can be controlled by cellular energy levels via the metabolic sensor AMP-activated Protein Kinase (AMPK) [9]. A reduced cellular ATP concentration results in elevation of AMP levels [10] that, in concert with upstream kinases leads to full activation of AMPK [11], [12], [13]. Activated AMPK coordinates an energy-conserving program by increasing cellular ATP production and reducing ATP consumption by shutting down energy intensive processes such as mTOR-dependent protein translation [9], [14]. AMPK inhibits mTOR via phosphorylation and activation of the Tuberous Sclerosis Complex (TSC) as well as directly phosphorylating the RAPTOR subunit of mTORC1 [15] (Fig. 1A).

The anti-diabetic drug and mitochondrial complex-1 toxin metformin is a potent activator of AMPK and both AMP dependent and independent mechanisms such as Reactive Nitrogen Species generation have been implicated in AMPK activation by metformin [16], [17], [18]. The glucose analogue 2-deoxy-D-glucose (2DG) is also able to activate AMPK through glycolytic inhibition [19] and we recently showed that 2DG administration suppresses epileptogenesis in the kindling model [20].

It is not known how the mTOR pathway in neurons, and thus L-LTP expression, is controlled by neuronal metabolism. Given that sustained LTP requires mTOR signaling, that mTOR is under metabolic control via AMPK, and previous reports suggest a link between energy metabolism and LTP [21], [22], [23], we reasoned that energy metabolism could regulate L-LTP via AMPK. Here we demonstrate that the energy sensor AMPK controls hippocampal L-LTP and provide evidence that this control is exerted through mTOR signaling.

## Methods

All procedures were performed with the approval of the University of Wisconsin-Madison School of Medicine and Public Health Institutional Animal Care and Use Committee and according to national guidelines and policies.

### Electrophysiology

All electrophysiology was performed on 4–6 wk old C57BL/*6* mice. Immediately after euthanasia the brain was removed from the skull and submerged in ice cold cutting solution (CS) [in mM]: 110 sucrose, 60 NaCl, 3 KCl, 1.25 NaH_2_PO_4_, 28 NaHCO_3_, 0.5 CaCl_2_, 7 MgCl_2_, 5 glucose, 0.6 ascorbate). The hippocampi were sectioned transversely in a Vibratome (St. Louis, MO) into 400 µM slices immersed in ice-cold CS. Slices were allowed to recover for 45 min at room temperature (RT) in 50∶50 CS: artificial cerebrospinal fluid (ACSF) [in mM]: 125 NaCl, 2.5 KCl, 1.25 NaH_2_PO_4_, 25 NaHCO_3_, 2 CaCl, 1 MgCl_2_, 25 glucose), and a further 45 min at RT in 100% ACSF before being transferred to an interface chamber (Fine Science Tools, Foster City, CA) bathed in 100% ACSF (1 mL/min) at 32°C (TC-324B, Warner Instrument Corporation, Hamden, CT) for 2 hours prior to treatment. All solutions were carb-oxygenated (95/5, O_2_/CO_2_). Enameled bipolar platinum-tungsten (92∶8 Pt∶Y) stimulating electrodes were placed along the Schaeffer-Collateral pathway. Field EPSPs were recorded from CA1 *stratum radiatum*, with ACSF-filled recording electrodes (5 MΩ). Baseline synaptic transmission was assessed for each individual slice by applying gradually increasing stimuli (0.5 V–15 V, 25 nA–1.5 µA, A-M Systems model 2200 stimulus isolator, Carlsborg, WA) to determine the input∶output relationship. All subsequent experimental stimuli were 50% of the intensity of the maximum evoked fEPSP slope (i.e. PPF, HFS, TBS). Paired-pulse facilitation was performed prior to the induction of LTP. PPF consisted of an initial single stimulus to the Schaeffer Collateral bundle followed by a second stimulus of equal magnitude. This paradigm was repeated with increasing time intervals between the two pulses. fEPSP slope measurements from the second pulse were plotted as a percentage of initial slope. LTP was induced with either high frequency stimulation (4 stimulations of 100 Hz each lasting for 1 second) or theta burst stimulation applied to the Schaeffer-collaterals and fEPSPs were measured in *stratum radiatum*. Theta burst stimulation consisted of 10 bursts/train, and 3 trains/stimulus with a 20 second intertrain interval. Each burst contained 4 stimulations at 100 Hz with an interburst interval of 200 msec. Synaptic efficacy was continually monitored (0.05 Hz). Every 2 min sweeps were averaged; the fEPSP's were amplified (A-M Systems model 1800), digitized (Digidata 1322B, Molecular Devices, Sunnyvale, CA) and then analyzed (pClamp, Molecular Devices). Two-way ANOVA (drug and time) with repeated measures (mixed model) and Bonferroni posttests were used for statistical analysis for drug effect over all points.

### Tissue Homogenization for Biochemistry

Following drug application and/or stimulation, slices were flash frozen in eppendorf tubes on dry ice. Slices were subsequently lysed with RIPA buffer (50 mM Tris, 150 mM NaCl, 1% nonidet P-40, 0.5% sodium deoxycholate, 0.1% SDS) + mammalian protease inhibitor (1∶100, Sigma, St. Louis, MO) + phosphatase inhibitors [in mM] (10 NaF, 2 Na Vanadate, 4 Na pyrophosphate, 10 β-glycerophosphate). Slices were then triturated with a 28.5 gauge insulin syringe to shear up DNA. Lysates were spun down at 12 krpm for 30 min and supernatants kept and quantified using the DC Protein Assay (Bio-Rad, Hercules, CA). Appropriate amounts of 5X loading buffer (0.5 M Tris, 10% SDS, 50% glycerol, 10 mM EDTA,1% β-mercaptoethanol) were added to protein extracts and boiled at 95°C for 3 min. All solutions were diluted with Milli-Q water (Milli-Q UF Plus, Millipore, Bedford, MA).

### Western Blotting

Protein extracts were loaded at 30 µg/lane in gradient (4–20%) tricine gels (Pierce Biochem, Rockford, IL) and resolved with standard electrophoresis in HEPES buffer (100 mM Tris, 100 mM HEPES, 0.1% SDS) and transferred overnight at 4°C onto PVDF membranes (Millipore, Bedford, MA) with tris-glycine buffer (20 mM Tris, 1.5 M glycine). Membranes were blocked with Tris-buffered salt solution with Tween-20 (TBST; 20 mM Tris pH 7.6, 150 mM NaCl, 0.1% Tween-20) and 5% milk fat for 1 hr prior to addition of primary antibody. To optimize binding, primary antibodies were either diluted in 5% milk fat or TBST. p-AMPK (Thr172) (1∶1000 overnight at 4°C in TBST, Cell Signaling, Danvers, MA), p-p70S6K (Thr389) (1∶1000 overnight at 4°C in TBST, Cell Signaling), p-rpS6 (Ser235/236) (1∶1000 overnight at 4°C in TBST, Cell Signaling), total-p70S6K (1∶1000 overnight at 4°C in TBST, Cell Signaling), total-rpS6 (1∶1000 overnight at 4°C in TBST, Cell Signaling), βIII tubulin (1∶10,000 for 1 hr at RT in milk, Promega, Madison, WI), actin (1∶10,000 for 1 hr at RT in milk, Millipore). Membranes were incubated for 1 hr in horseradish peroxidase-conjugated goat anti-rabbit IgG or goat anti-mouse IgG secondary antibodies (1∶10,000) (Santa Cruz Biotech All solutions were diluted with Milli-Q water (Milli-Q UF Plus, Millipore, Bedford, MA). Protein bands were detected using SuperSignal West Femto ECL reagent (Pierce Biochem) and visualized using Kodak Image Station 2000R and Kodak 1D Image Analysis software, which was also used to quantify protein bands and the two-tailed Student's T-test was employed for statistical analysis.

### Immunofluorescence and Microscopy

Tissue analysis was performed on 400 µm hippocampal slices following incubation in either ACSF or ACSF plus 10 mM 2DG. Slices were incubated overnight in a fixative solution containing 4% formaldehyde (freshly depolymerized from paraformaldehyde; Sigma, St. Louis, MO) in 0.1 M phosphate buffer, pH 7.4 (PB). Slices were removed from fixative, rinsed in PB and cryoprotected in 50% PB/50% cryoprotectant with 20% sucrose and 5% glycerol for 1 hour, followed by 100% cryprotectant with 20% sucrose and 5% glycerol for 1 hour. Slices were frozen with dry ice and sectioned on a sliding microtome in the coronal plane at 30 µm thickness. Sections were transferred to PB at 4°C (with 0.01% sodium azide if stored for more than 2 days).

Anti-phospho-AMPKα (Thr172) was purchased from Santa Cruz (sc-33524, lot F0209). Anti-NeuN was purchased from Millipore (MAB377, lot LV1573084). Sections from both septal and temporal hippocampus were prepared for light microscopy.

Frozen sections were rinsed in 0.01 M phosphate buffered saline (PBS) with 0.1% saponin (product S7900; Sigma Aldrich) and 2% bovine serum albumin (BSA; Calbiochem, La Jolla, CA), blocked in the same buffer with 20% normal goat serum for 45 minutes, incubated overnight in primary antiserum to p-AMPKα (1∶500) and primary antiserum to NeuN (1∶1000) with 0.1% normal goat serum, washed in buffer, incubated for 2 hours in 1∶500 AlexaFluor 488 goat anti-rabbit IgG (product A11034, lot 461250 from Molecular Probes, Eugene, OR) and 1∶500 AlexaFluor 594 goat anti-mouse IgG (product A11032, lot 419361 from Molecular Probes, Eugene, OR) in PBS with 0.1% saponin and 2% BSA, rinsed with PB and mounted with SlowFade Light Antifade Kit (product S2828, lot 54616A from Molecular Probes, Eugene, OR).

Three “positive” controls were performed to minimize the possibility of artifactual staining. 1) p-AMPKα and NeuN were visualized with both immunofluorescence and immunoperoxidase reaction methods. 2) Dilution series were carried out to obtain optimal staining dilutions. 3) p-AMPKα and NeuN antisera were visualized on transcardial perfused tissue fixed with 4% formaldehyde. No discrepancies were observed in the pattern of label in these comparisons. For a “negative” control, immunoreactions were run without primary antisera. No label was observed with this control.

Light microscopic imaging was performed with a digital camera (Spot II; Diagnostic Instruments, Sterling Heights, MI) on a Nikon E600 Eclipse epifluorescent microscope with x2–60 planapochromatic objectives and a standard FITC filter cube (FITC; EX 465–495 nm; DM 505 nm; BA 515–555 nm) and TRITC filter cube (TRITC; EX 528–553 nm; DM 565 nm; BA 600–660 nm). Images were obtained with the x40 objective. Fluorescent images were acquired at an initial 36-bit tone scale and saved as 16-bit files. Light microscopic images were prepared for reproduction in Adobe Photoshop 7.0 with minimal adjustments in the tone scale, contrast, hue and subsequent sharpening with the unsharp mask algorithm.

## Results

### AMPK Activation in the Hippocampus Represses mTOR Signaling

To test the hypothesis that metabolism can control L-LTP via the action of AMPK on mTOR signaling, we first tested whether energy stress could activate AMPK in the hippocampus. Hippocampal slices were incubated in either ACSF or ACSF containing the glycolytic inhibitor 2-deoxy-D-glucose (2DG) for 30 minutes, and AMPK activation was assessed by measuring phosphorylated AMPKα1/2 (p-AMPK). Exposure to 10 mM 2DG (a concentration that allows for competitive inhibition of transporters and kinases of 25 mM glucose) resulted in a 2 to 3 fold induction of p-AMPK compared to control slices (Fig. 1B and 1C). This AMPK activation correlated with a 2 fold reduction in ATP levels (Fig. 1D). The anti-diabetic drug metformin is a potent activator of AMPK in other tissues: Figures 1B and 1C show that 5 µM metformin and the related molecule phenformin are also able to activate AMPK 2 to 3 fold in the hippocampus. To determine whether AMPK activation occurs in CA1 dendrites (the site of mTOR activation upon LTP induction [6]) we visualized p-AMPK using immunofluorescence on mouse hippocampal slices. Figure 1E shows that p-AMPK immunoreactivity is present both in the dendrites of the stratum radiatum (SR) and cell bodies of the pyramidal layer in CA1. Addition of 2DG results in a transition from a punctate pattern to a smoother and brighter pattern in the dendrites (compare panels 1 and 4). There is also a marked increase in cell body staining in the presence of the AMPK activator. The increased diffuse staining throughout the SR in the presence of 2DG may be due to activation of AMPK in the glial compartment. However, the observed activation of AMPK in the dendrites demonstrates that hippocampal neurons contain the necessary mechanisms to activate AMPK upon administration of known AMPK inducers.

**Figure 2:**
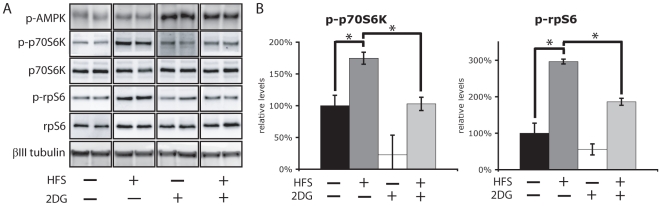
AMPK activation represses the mTOR pathway. 10 mM 2DG inhibits the hippocampal mTOR pathway. A) HFS was delivered to the Schaeffer Collateral pathway of slices that had been incubated in the presence or absence of 10 mM 2DG for 30 minutes. Slices were then subjected to western blot analysis using anti-phospho-Thr389-p70S6K, anti-p70S6K, anti-phospho-Ser235/236-rpS6 or anti-rps6 antibody. Representative western blots of duplicate lanes and quantification (B) of 12 samples per condition are shown. Error bars show standard error of the mean (s.e.m). *p<0.05.

The mTOR pathway is activated in CA1 within 5 minutes of High Frequency Stimulation (HFS) and is necessary for expression of L-LTP [6], [24]. Activation of mTOR can be monitored by phosphorylation of its downstream targets and so we looked at the mTOR substrate p70-S6Kinase (p70S6K) and its substrate ribosomal protein S6 (rpS6) due to their established role directly downstream of the mTOR kinase. We confirmed the observations of Tsokas *et al.* that HFS of the Schaeffer Collaterals induces phosphorylation of the mTOR cascade components p70S6K and rpS6 [6] as judged by western blotting of protein from stimulated or un-stimulated slices (Fig. 2). Consistent with the hypothesis that AMPK activation suppresses mTOR signaling in the hippocampus, high frequency stimulation failed to induce rpS6 or p70S6K phosphorylation in the presence of 2DG.

### AMPK Activation Prevents L-LTP Expression

**Figure 3:**
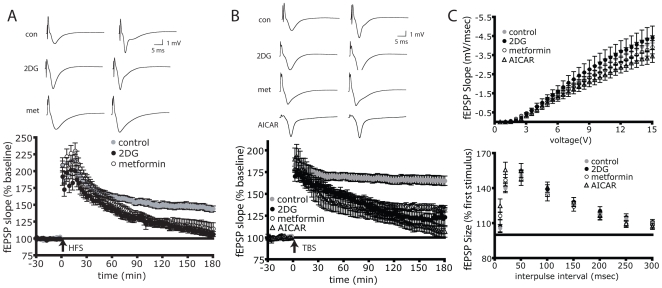
AMPK activation inhibits L-LTP expression. A) AMPK activation inhibits LTP induced by HFS. 10 mM 2DG (n = 10) reduces L-LTP to 10% of control (n = 20) (p = 0.034). 5 µM metformin (n = 8) reduces L-LTP to 40% of control (p = 0.028). B) AMPK activation inhibits LTP induced by TBS. 10 mM 2DG reduces L-LTP (p = 0.022, n = 7) to 30% of control (n = 13), 5 µM metformin reduces L-LTP (p = 0.042, n = 12) to 51% of control. 1 mM AICAR reduces L-LTP (p = 0.0025, n = 8) to 11% of control C) 2DG, metformin and AICAR do not have an effect on basic synaptic transmission. Top: input-output relationships for Schaeffer collateral stimulation and fEPSP slope measured in the presence of ACSF (n = 27), 10 mM 2DG (n = 14), 5 µM metformin (n = 17) or 1 mM AICAR (n = 8). Bottom: Paired Pulse Facilitation is not affected by the presence of 10 mM 2DG (n = 11), 5 µM metformin (n = 17) or 1 mM AICAR (n = 8) compared to ACSF alone (n = 14). Results are plotted as the ratio of fEPSP slopes (2^nd^ stimulus/1^st^ stimulus X100) as a function of interpulse interval (0–300 msec). *p = 0.0002. A and B) Inset: representative fEPSP traces shown were taken 4 minutes prior and 180 minutes after stimulation. Error bars show s.e.m.

Given the necessity of mTOR signaling for L-LTP expression, we predicted that AMPK activation should prevent L-LTP expression. To test this hypothesis, we initially tested the effects of the AMPK activator 2DG on LTP that was induced using 2 different mTOR dependent paradigms: HFS and Theta Burst Stimulation (TBS) [25]. HFS or TBS was delivered between CA1 and CA3 in the Schaeffer Collateral bundle in the presence or absence of 2DG and field excitatory post-synaptic field potentials (fEPSPs) were recorded in the stratum radiatum of CA1. Following either HFS or TBS, the induction step of LTP was indistinguishable between 2DG treated and control slices (Fig. 3A and 3B, respectively), consistent with this step being mTOR independent [7]. However 60 minutes post-stimulation the 2DG-treated slices failed to maintain L-LTP, which falls to 10% of untreated over the course of 3 hours in the HFS paradigm and 30% in the TBS paradigm.

We reasoned that if 2DG inhibits L-LTP via AMPK activation, then other AMPK activators should also inhibit L-LTP. Therefore we tested whether the AMPK activators metformin [16] or 5-AminoImidazole-4-CarboxAmide Ribonucleoside (AICAR) [26] could also inhibit L-LTP induced by TBS or HFS. Figure 3A and 3B show that similar to 2DG, 5 µM metformin suppressed L-LTP but did not impact LTP induction. Figure 3B also shows that AICAR inhibits L-LTP induced by TBS. Therefore, three independent and structurally unrelated activators of AMPK eliminated L-LTP that was elicited by two different stimulation paradigms. Importantly, 2DG, AICAR and metformin did not affect synaptic transmission *per se* because there was no significant difference in the input/output relationship of the fEPSP slope magnitude as a function of stimulus voltage when compared to ACSF alone (Fig. 3C, top; AICAR vs. control, p = 0.223; 2DG vs. control, p = 0.702; metformin vs. control, p = 0.573).

If AMPK activation works to suppress mTOR signaling to inhibit L-LTP, then the effects of AMPK activation would be predicted to be postsynaptic given that LTP induction results in dendritic rather than axonic mTOR activation [6]. To test this we assessed whether 2DG, metformin or AICAR affected Paired-Pulse Facilitation (PPF) (a protocol widely used to assess a presynaptic component of a response to a stimulation or compound [27]). The PPF protocol consists of an initial stimulation followed by a second stimulation after a given interval. The second stimulus evokes a greater response (measured as fEPSP slope) than the initial stimulus due to residual Ca^2+^ in the presynaptic terminal which facilitates an increased amount of neurotransmitter release. If a compound inhibits release, there will be less neurotransmitter released initially, and therefore the second stimulus will elicit a greater release of neurotransmitter and thus a greater response. Figure 3C (bottom) shows that there was little difference in PPF between control slices or those administered drugs (AICAR vs. control, p = 0.2061; 2DG vs. control, p = 0.6006; metformin vs. control, p = 0.9076). This result is consistent with the hypothesis that AMPK activation acts to inhibit mTOR signaling to suppress L-LTP.

**Figure 4:**
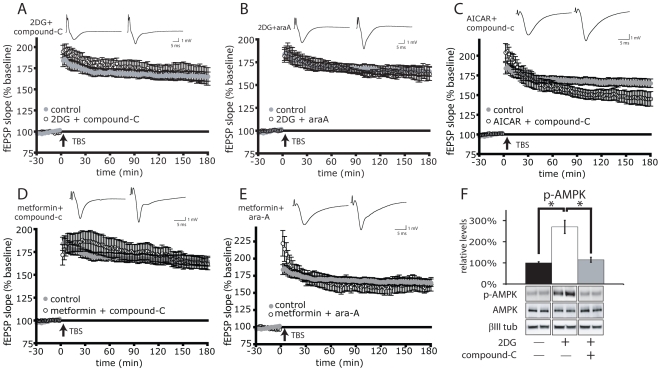
AMPK inhibition rescues L-LTP expression. AMPK inhibitors prevent TBS-induced L-LTP loss in the presence of 2DG, metformin or AICAR. A) 1 µM compound-C (n = 9) or B) 100 µM araA (n = 6) prevent 10 mM 2DG-mediated loss of L-LTP. C) 1 µM compound-C prevents 1 mM AICAR-mediated loss of L-LTP (n = 6). D) 1 µM compound-C (n = 9) or E) 100 µM araA (n = 6) prevents 5 µM metformin-mediated loss of L-LTP. Control data from Fig. 3B is reproduced in Fig. 4A and Fig. 4B for comparison. A–E) Inset: representative fEPSP traces shown were taken 4 minutes prior and 180 minutes after stimulation. F) 1 µM compound-C abolishes 10 mM 2DG-mediated AMPK activation. Slices were incubated in ACSF (n = 8), 10 mM 2DG (n = 5) or both 10 mM 2DG and 1 µM compound-C (n = 6) for 30 minutes, subjected to western blotting and quantified as in Fig. 1A. *p = 0.0002. Error bars show s.e.m.

If AMPK activation is necessary for 2DG, metformin or AICAR to inhibit L-LTP, then addition of an AMPK inhibitor should prevent 2DG, metformin or AICAR from repressing L-LTP. Figure 4 shows that in the presence of the potent AMPK inhibitor compound-C [16], 2DG treatment failed to activate AMPK (Fig. 4F). In the presence of compound-C, 2DG had no effect on TBS-induced LTP expression (Fig. 4A). AMPK inhibition using a structurally unrelated molecule, ara-A [28] also prevented 2DG from repressing L-LTP expression (Fig. 4B). LTP suppression by metformin was similarly inhibited by either compound-C or ara-A (Fig. 4D and 4E respectively). Finally, LTP suppression by the AMPK activator ACIAR was prevented by compound-C (Fig. 4C). These data with three AMPK activators and two inhibitors support the hypothesis that L-LTP expression in the hippocampus is under metabolic control via the metabolic sensor AMPK.

**Figure 5:**
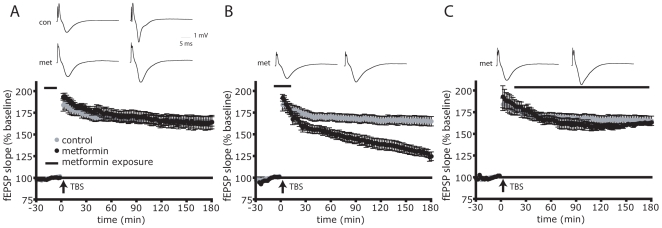
AMPK activation suppresses L-LTP within a time-restricted window. A) 5 µM metformin was added 20 minutes prior to TBS and washed out immediately prior to TBS (n = 8). L-LTP at 180 minutes post TBS was equal to control (n = 17), B) metformin was added immediately prior to TBS and washed out immediately after TBS (n = 8). L-LTP was reduced to 30% of control (p = 0.0172). C) metformin was added 5 min after stimulation for the duration of the experiment (n = 5). L-LTP at 180 minutes was indistinguishable from control. Control data in Fig. 5A is reproduced in Fig. 5B and Fig. 5C for comparison. Inset: Representative fEPSP traces shown were taken 4 minutes prior to and 180 minutes after TBS. Error bars show s.e.m.

L-LTP inducing stimuli (e.g. TBS and HFS) rapidly activate postsynaptic translational machinery in order to produce key proteins involved in the induction and maintenance of LTP [5], [29]. In the presence of protein synthesis inhibitors, LTP-inducing stimuli produce short-term potentiation, yet fail to produce lasting LTP [30], [31]. However, LTP is unaffected if protein synthesis inhibitors are added just after the tetanus [24]. Late-Phase LTP elicited by either HFS or TBS is eliminated by the mTOR inhibitor rapamycin. Further, Cammalleri *et al.* demonstrated a critical time window of rapamycin sensitivity in inhibition of L-LTP: exposure to rapamycin during tetanic stimulation is sufficient to prevent expression of LTP two hours later [24]. If AMPK activation represses mTOR signaling to inhibit L-LTP, then metformin exposure solely during the stimulation protocol should be sufficient to prevent L-LTP expression. Figure 5B shows that metformin administration during TBS followed by wash-out immediately after the stimulation paradigm generates a loss of L-LTP identical to that seen in the continued presence of metformin (Fig. 3B). To show that this was not due to persistence of metformin after wash out, metformin was added and washed out prior to stimulation, with no effect on L-LTP (Fig. 5A). Also, addition of metformin after stimulation for the remaining three hours had no significant effect on L-LTP (Fig. 5C). This result suggests that a critical period of metformin/AMPK sensitivity exists during the induction phase of LTP, which overlaps with the mTOR dependent period defined by Cammalleri *et al*.

**Figure 6:**
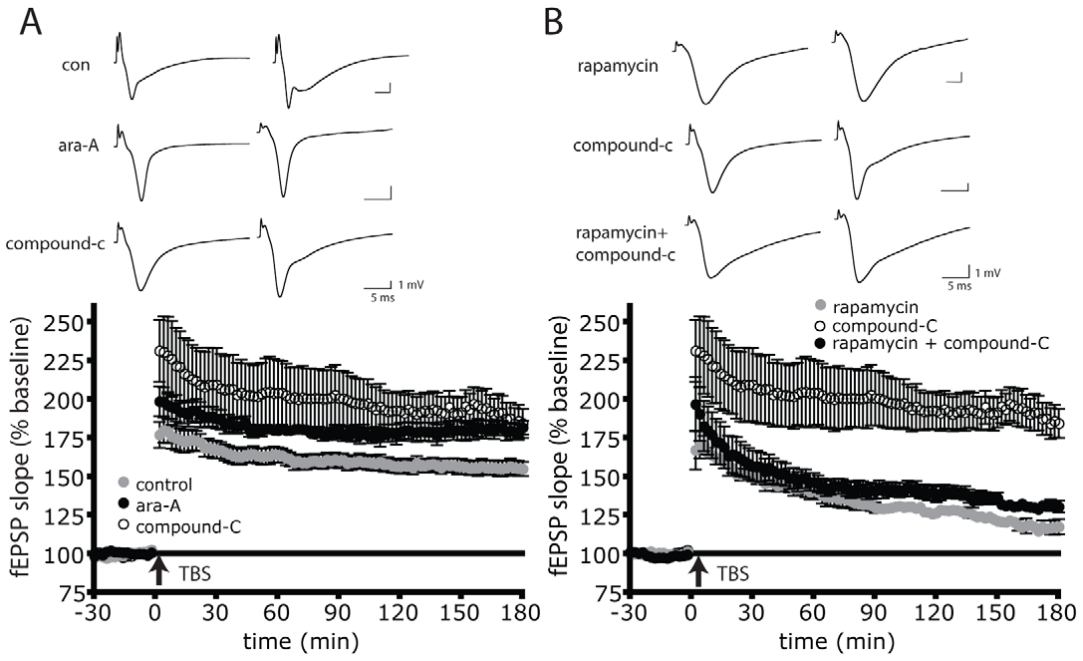
AMPK regulation of L-LTP is rapamycin sensitive. A) 1 µM compound-C (n = 8, p = 0.0069) or 100 µM ara-A (n = 8, p = 0.0045) results in heightened potentiation after TBS compared to ACSF alone (n = 14). B) 1 µM rapamycin results in suppression of L-LTP to 33% of control (n = 4, p = 0.0097). 1 µM compound-C in the presence of rapamycin fails to significantly enhance L-LTP above rapamycin alone (n = 9, p = 0.2706). Control data in Fig. 6A is reproduced in Fig. 6B for comparison. Inset: Representative fEPSP traces shown were taken 4 minutes prior to and 180 minutes after TBS. Error bars show s.e.m.

To explore the role of mTOR in AMPK modulation of LTP we took advantage of the observation that treating slices with the AMPK inhibitors compound-C or ara-A results in hyper-potentiation (Fig. 6A). If AMPK inhibition results in heightened LTP due to de-repression of mTOR signaling then in the presence of the mTOR inhibitor rapamycin, compound-C should have no effect. Figure 6B shows that as reported by others, rapamycin eliminates L-LTP induced by TBS. Compound-C fails to elevate LTP in the presence of rapamycin suggesting that AMPK modulation of LTP requires mTOR signaling. In aggregate, these physiological measurements, together with the molecular analyses suggest a model whereby AMPK activation inhibits maintenance of LTP at least in part, through suppression of the mTOR pathway.

## Discussion

The work presented here suggests that LTP is under metabolic control via the energy sensor AMPK. This is supported by the observation that three structurally distinct activators of AMPK (2DG, metformin and AICAR) suppress L-LTP expression in two different LTP paradigms (HFS and TBS) (Fig. 3). Secondly, LTP suppression by 2DG, metformin and AICAR can be overcome by two inhibitors of AMPK that are structurally and mechanistically distinct (compound-C and ara-A) (Fig. 4). It is likely that the suppressive effects of AMPK activation work at least in part through the mTOR pathway. This hypothesis is supported by the observation that the AMPK activator 2DG suppresses phosphorylation and activation of the downstream mTOR pathway components, p70S6K and rpS6 upon High Frequency Stimulation (Fig. 2). Secondly, rapamycin prevents the enhancement of LTP by the AMPK inhibitor compound-C suggesting that AMPK is upstream of mTOR in regulating LTP (Fig. 6). Thirdly, AMPK activators fail to alter Paired Pulse Facilitation, which suggests a post-synaptic mode of action, consistent with the site of mTOR activity [7], [32]. Fourthly, AMPK activation restricted to the stimulation period is sufficient to prevent L-LTP expression three hours later, an observation consistent with the time period required for mTOR activity to maintain L-LTP [24]. Data presented here connects AMPK to neuronal mTOR signaling thereby linking energy and metabolic status to LTP. In conjunction with published work describing the role of mTOR in LTD [33], our work suggests that neuronal plasticity in general is under the direct control of cellular energy metabolism through AMPK.

Late-Phase LTP requires MAPK/ERK and PI3/AKT signaling that activates the mTOR pathway and dendritic protein translation [6]. L-LTP also has a requirement for gene transcription including a critical role for the cAMP Response Element Binding protein (CREB) activity and BDNF expression [5], [34], [35]. Interestingly CREB and TORC1 signaling is known to be modulated by AMPK in peripheral tissue [13], [36], [37], suggesting that AMPK may also work through CREB to control plasticity.

In addition to positively regulating protein synthesis, mTORC1 contributes to a variety of other cellular processes, some of which have been linked to neuronal function. mTOR activity impacts mitochondrial function by increasing oxidative capacity and promoting the transcription of key components of the electron transport chain [38], [39]. Consequently, hyperactive mTOR signaling increases the generation of reactive oxygen species (ROS), which are required for LTP [40]. Hence it is possible that inhibition of mTOR signaling by AMPK activation could suppress LTP via a reduction in ROS production.

Additionally, mTOR acts to inhibit glycogen synthase kinase 3β (GSK3β) signaling [41]. Given the potential role of GSK3β in enhancing LTP [42], activation of GSK3β via mTOR inhibition may also contribute to loss of LTP in our system. Nevertheless, our results suggest that the negative regulation of mTOR signaling by AMPK links energy sensing to plasticity.

Several reports have demonstrated an increased fEPSP when glucose is replaced by 2DG for some time period [43], [44], [45]. In these studies, 2DG application results in a decreased fEPSP slope, which is reversed upon switching back to normal ACSF leading to potentiation. Zhao *et al*. reported that 2DG suppresses synaptic transmission through release of adenosine and activation of pre-synaptic adenosine receptors [46]. This would imply a pre-synaptic mode of action for 2DG and would result in an altered PPF (compared to control) at CA1. In our hands we do not see an effect on PPF by 2DG or metformin, which is consistent with the hypothesis that the effects of AMPK are primarily post-synaptic. It is therefore unlikely that the negative regulation of LTP by AMPK works through adenosine release. The discrepancy between the results reported by Zhao *et al*. and our work is most likely due to the fact that Zhao *et al*. replace glucose with 2DG in their ACSF. We administer 10 mM 2DG in the presence of 25 mM glucose in ACSF and this results in a tempering of ATP production to 50% of ACSF alone (Fig. 1D), yet does not alter baseline synaptic responses out to four hours of recording (data not shown). This major difference in paradigm most likely explains why Zhao *et al*. observe a pre-synaptic effect by 2DG via adenosine release whereas our 2DG effects are post-synaptic, as judged by the lack of change in PPF.

Our findings that AMPK controls LTP may provide a cellular and molecular basis for the observations that increased glucose availability enhances learning and memory [47], [48], [49], [50]. During exploratory behavior, plasma brain glucose levels drop. When glucose levels are maintained with glucose injections, rats perform better in a passive avoidance test compared to controls. Rats injected with glucose immediately after training in this same avoidance test showed enhanced retention of memory 24 hours later compared to controls. However, injection 1 hour after training had no effect on retention [47]. Similarly, intra-amygdala glucose injection helped the extinction of conditional place preference only when glucose administration occurred immediately after training but not two hours later [51]. The ability of glucose to impact learning and memory tasks implies that learning and memory mechanisms are under metabolic control.

The inability of glucose to improve behavioral outcomes when administered 1–2 hours after training as compared to directly after is consistent with our findings that AMPK activation must occur during the stimulation period to inhibit L-LTP. mTOR activity is not continuously required after LTP induction in order to sustain L-LTP but instead is necessary only during the stimulus period [24]. This suggests that *de novo* protein synthesis upon induction generates products required to maintain LTP in the absence of continued stimulation. In keeping with this model, our data shows that a narrow time window exists during which AMPK activation is sufficient to suppress L-LTP: the presence of metformin just during the stimulus period followed by wash-out prevents LTP maintenance whereas addition of metformin immediately after stimulation for the duration of the experiment has no effect on L-LTP (Fig. 5). This result suggests that acute suppression of mTOR via AMPK activation during the short induction phase of LTP prevents expression of chronic L-LTP.

It is tempting to consider that AMPK modulation of L-LTP *in vitro* reflects AMPK modulation of learning and memory *in vivo*. Indeed Dash *et al.* demonstrated that activation of neuronal AMPK with hippocampal injections of AICAR reduced long-term memory and this was associated with reduced phosphorylation of mTOR cascade components. Importantly, glucose injection directly into the rat hippocampus improved long-term spatial memory in the Morris Water Maze and this was associated with decreased AMPK phosphorylation/activation and increased activity of the mTOR cascade [52]. The fact that AMPK activity can be reduced in the hippocampus and result in heightened long-term memory demonstrates that under physiological conditions, latent AMPK activity tempers long-term memory mechanisms. This notion is consistent with the observation that application of AMPK inhibitors (compound-C and ara-A) in the absence of AMPK agonists result in heightened LTP (Fig. 6). Therefore we argue that metabolic regulation of LTP and long-term memory is not only important under pathological conditions where energy stress may be present but is also important under physiological conditions. The results presented here along with published work supports a model whereby neuronal energy status controls LTP maintenance through the action of AMPK on mTOR dependent dendritic protein translation.

The possibility of controlling plasticity through AMPK may offer a route to therapeutic intervention in certain neurological disorders. Tuberous Sclerosis is caused by mutations in the TSC1 or TSC2 gene and is often associated with autism, mental retardation and epilepsy [53], [54]. Loss of TSC function results in heightened mTOR activity and inappropriate LTP induction upon a single high frequency stimulation in slice experiments [55]. Importantly, addition of the mTOR inhibitor rapamycin reverses many of the behavioral deficits in TSC mutant mice and prevents inadvertent maintenance of LTP. These intriguing results indicate that pharmacological suppression of heightened mTOR signaling in Tuberous Sclerosis patients might be of therapeutic value. Our work suggests that the widely used anti-type II diabetes drug metformin can suppress mTOR signaling through activation of AMPK in the hippocampus. Metformin is used by around 35 million people in the U.S alone, is able to cross the blood-brain barrier and has few contra-indications when prescribed appropriately [16], [56], [57]. The absence of a functional TSC1/2 complex prevents AMPK from inhibiting mTOR signaling through phosphorylation of TSC2, however work by Gwinn *et al.* shows that AMPK can also suppress mTOR through the targeting of RAPTOR [15]. Therefore, heightened mTOR signaling in TSC patients could feasibly be attenuated therapeutically through the action of metformin on AMPK and RAPTOR.

We show that the glycolytic inhibitor 2DG is a potent activator of AMPK, prevents stimulation-induced mTOR activation, and suppresses L-LTP in an AMPK-dependent manner. These properties are of particular interest because we recently showed that 2DG retards epileptogenesis in the rat electrical kindling model of temporal lobe epilepsy [20]. This modulation of activity dependent plasticity was associated with reduced BDNF and TrkB expression and increased recruitment of the metabolic sensor CtBP to the transcriptional repressor NRSF/REST [58], [59]. BDNF is an upstream component of the mTOR cascade and is sufficient to enhance potentiation at CA1 synapses in a rapamycin sensitive manner [7]. Therefore mTOR dependent LTP may be regulated by metabolism via a transcription dependent mechanism through CtBP and via a post-translational mechanism through AMPK. It will be interesting to test whether metformin, working through AMPK has the same effects as 2DG working through CtBP in retarding kindling and epileptogenesis. In summary, we show that plasticity is under metabolic control through the activity of the master energy regulator, AMPK.
